# Clinical Use of Bedside Portable Low-field Brain Magnetic Resonance Imaging in Patients on ECMO: The Results from Multicenter SAFE MRI ECMO Study

**DOI:** 10.21203/rs.3.rs-3858221/v1

**Published:** 2024-01-16

**Authors:** Sung-Min Cho, Shivalika Khanduja, Christopher Wilcox, Kha Dinh, Jiah Kim, Jin Kook Kang, Ifeanyi David Chinedozi, Zachary Darby, Matthew Acton, Hannah Rando, Jessica Briscoe, Errol Bush, Haris I Sair, John Pitts, Lori R Arlinghaus, Audrey-Carelle N Wandji, Elena Moreno, Glenda Torres, Bindu Akkanti, Jose Gavito-Higuera, Steven Keller, HuiMahn A Choi, Bo Soo Kim, Aaron Gusdon, Glenn JR Whitman

**Affiliations:** Johns Hopkins University School of Medicine; Johns Hopkins Hospital: Johns Hopkins Medicine; Johns Hopkins Hospital: Johns Hopkins Medicine; UTHSC: The University of Texas Health Science Center at Houston; Johns Hopkins Hospital: Johns Hopkins Medicine; Johns Hopkins Hospital: Johns Hopkins Medicine; Johns Hopkins Hospital: Johns Hopkins Medicine; Johns Hopkins Hospital: Johns Hopkins Medicine; Johns Hopkins Hospital: Johns Hopkins Medicine; Johns Hopkins Hospital: Johns Hopkins Medicine; Johns Hopkins Hospital: Johns Hopkins Medicine; Johns Hopkins Hospital: Johns Hopkins Medicine; Johns Hopkins Hospital: Johns Hopkins Medicine; Hyperfine Inc; Hyperfine Inc.; UTHSC: The University of Texas Health Science Center at Houston; UTHSC: The University of Texas Health Science Center at Houston; UTHSC: The University of Texas Health Science Center at Houston; UTHSC: The University of Texas Health Science Center at Houston; UTHSC: The University of Texas Health Science Center at Houston; Johns Hopkins Hospital: Johns Hopkins Medicine; UTHSC: The University of Texas Health Science Center at Houston; Johns Hopkins Hospital: Johns Hopkins Medicine; UTHSC: The University of Texas Health Science Center at Houston; Johns Hopkins Hospital: Johns Hopkins Medicine

**Keywords:** Portable MRI, ultra-low-field MRI, ECMO, critical care, acute brain injury, ABI

## Abstract

**Purpose:**

Early detection of acute brain injury (ABI) is critical for improving survival for patients with extracorporeal membrane oxygenation (ECMO) support. We aimed to evaluate the safety of ultra-low-field portable MRI (ULF-pMRI) and the frequency and types of ABI observed during ECMO support.

**Methods:**

We conducted a multicenter prospective observational study (NCT05469139) at two academic tertiary centers (August 2022-November 2023). Primary outcomes were safety and validation of ULF-pMRI in ECMO, defined as exam completion without adverse events (AEs); secondary outcomes were ABI frequency and type.

**Results:**

ULF-pMRI was performed in 50 patients with 34 (68%) on venoarterial (VA)-ECMO (11 central; 23 peripheral) and 16 (32%) with venovenous (VV)-ECMO (9 single lumen; 7 double lumen). All patients were imaged successfully with ULF-pMRI, demonstrating discernible intracranial pathologies with good quality. AEs occurred in 3 (6%) patients (2 minor; 1 serious) without causing significant clinical issues.

ABI was observed in ULF-pMRI scans for 22 patients (44%): ischemic stroke (36%), intracranial hemorrhage (6%), and hypoxic-ischemic brain injury (4%). Of 18 patients with both ULF-pMRI and head CT (HCT) within 24 hours, ABI was observed in 9 patients with 10 events: 8 ischemic (8 observed on ULF-oMRI, 4 on HCT) and 2 hemorrhagic (1 observed on ULF-pMRI, 2 on HCT).

**Conclusions:**

ULF-pMRI was shown to be safe and valid in ECMO patients across different ECMO cannulation strategies. The incidence of ABI was high, and ULF-pMRI may more sensitive to ischemic ABI than HCT. ULF-pMRI may benefit both clinical care and future studies of ECMO-associated ABI.

## Introduction

The use of extracorporeal membrane oxygenation (ECMO) has dramatically increased in the past decade for patients with refractory cardiopulmonary failure.^1^ However, ECMO conveys an elevated risk of acute brain injury (ABI), such as ischemic stroke, intracranial hemorrhage (ICH), and hypoxic-ischemic brain injury.^2^ Early diagnosis of ABI appears important as it can gfluide clinicians regarding timely cessation and judicious resumption of anticoagulation therapy, mitigating the dire consequences of ECMO-associated ABI which is known to increase mortality by a factor of 2–3.^3,4^

Currently, the timing and the true prevalence of ABI are unknown because assessing neurological status in ECMO patients is challenging and often delayed owing to the patients’ critical illness complicated by the routine use of sedation, paralytics, and the lack of standardized neurological examination and assessment. Even if standardized neurological monitoring improves the detection of ABI,^2,5^ their persistent cardiopulmonary instability makes the timely diagnosis and management of ABI in these patients difficult.^5^ More than 30% of patients do not receive head CT (HCT) scans during ECMO support due to an inability to safely transport patients and the lack of readily available trained transport personnel.^5^ Even where possible, head CT has limited sensitivity for detecting hyperacute to acute ischemic ABI.^6^ The gold standard for diagnosing ABI, conventional magnetic resonance imaging (MRI), relies on strong magnetic fields (1.5–3T) which are incompatible with extracorporeal life support circuits and equipment due to safety concerns (heating, migration, and malfunction). In summary, an inability to obtain timely neuroimaging remains a significant barrier to effectively detecting and treating ABI in patients on ECMO.

Ultra-low-field (< 0.1T) portable MRI (ULF-pMRI) technology specifically addresses this issue, enabling clinically meaningful, sensitive imaging in the presence of ferromagnetic materials in complex clinical care settings, such as intensive care units (ICUs), without causing significant adverse events.^7^ By virtue of its markedly smaller magnetic field footprint compared to conventional MRI, this technology allows for the use of equipment that is not MR-compatible, even as close as a few feet of the system.^7^ ULF-pMRI has a reduced specific absorption rate which diminishes the heating of conductive devices and implants, and eliminates pulse sequence parameter constraints.^8^ Our phantom and animal studies have shown the safety and compatibility of pMRI with the ECMO circuit components, without deleterious sequelae of the magnetic forces or heating,^9^ a safety and efficacy supported by its use in a case series in adults supported by ECMO.^10^

Our current study aimed to determine the safety of ULF-pMRI and validate its diagnostic capability in ECMO patients in a multicenter prospective observational study. Furthermore, we aimed to investigate the frequency and types of ABI occurring during ECMO support, an evaluation unhindered by the logistics and danger of transporting these patients to off-unit imaging.

## Methods

The study was conducted following the Declaration of Helsinki and approved by the Institutional Review Boards of The Johns Hopkins Medicine (IRB00285716 approved on 08/10/2021) and the University of Texas-Houston (HSC-MS-22–0608 approved on 11/07/2022). Informed consent was obtained from a legally authorized representative as ECMO patients were unable to provide consent.

This study, conducted from August 2022 to November 2023, was a prospective observational study (SAFE MRI ECMO study: NCT05469139) performed at these two academic tertiary referral centers (Johns Hopkins Hospital and the University of Texas-Houston) with high ECMO volumes.

### Patient Population

Patients enrolled in the study were adults (≥ 18 years) supported with either venoarterial (VA) or venovenous (VV) extracorporeal membrane oxygenation (ECMO). Exclusion criteria were the well-known contraindications for conventional MRI (**Supplemental Table 1**).

### Study Procedure

Point-of-care MR exams were performed with a 64mT *Swoop*^®^ MR imaging system (Hyperfine, Inc., Guilford, CT; hardware versions 1.6 and 1.8 with software versions 8.6.1 and rc8.7). The MR system was moved into the patient’s room by a trained operator. All ICU equipment remained outside the 5-gauss line. Once the patient’s bed was aligned with the MR system’s transfer bridge, 4–6 trained people (physician leader, post-doctoral fellow or coordinator, respiratory therapist, perfusionist, and nurses) used the patient’s bed sheets to move the patient into the scanner using a lift-and-slide maneuver. Detailed explanations and safety protocols in patient and equipment positioning are described in our prior publication.^10^ T1-weighted, T2-weighted, fluid-attenuated inversion recovery (FLAIR), and diffusion-weighted imaging (DWI) with automatically calculated apparent diffusion coefficient (ADC) map sequences were acquired. All MR images were read by a board-certified neuroradiologist in each center (H.I.S.; J.G-H.), blinded to patients’ clinical information and neurological status.

When possible, pMRI was obtained within the first 72 hours of ECMO cannulation allowing comparison to a conventional head CT (HCT), the latter ordered as part of a standardized neuromonitoring protocol,^5^ performed within 24 hours of the pMRI exam.

The patient’s vital signs were monitored continuously and recorded every minute during the MR exam/image acquisition. In addition, ECMO flow, cannula position, and endotracheal tube position were monitored during the scan. Patient demographics, pre-ECMO comorbidities, severity of critical illness including Glasgow Coma Scale (GCS), ECMO cannulation strategy, ECMO duration, and survival to hospital discharge (mortality) were also recorded.

### Adverse Events and Stopping Rules

Adverse events (AEs) were monitored during the pMRI scan and defined as follows: i) a change in mean arterial pressure (MAP) of ± 20%; ii) a decrease in ECMO flow of > 10%; or iii) a decrease in peripheral oxygen saturation (SpO_2_) of > 10%. AEs were further categorized by severity, as minor or serious, and by relation to the pMRI exam, related or unrelated, as assessed by the study team (S.-M.C., A.G., G.J.W., and K.D.). In the case of a serious AE, MR exams were paused to assess the cause and safety of resuming the exam.

### Outcomes

Primary outcomes were the safety of using ULF-pMRI in patients during ECMO support, defined as completion of pMRI exam without serious adverse events (AEs), and validation of useful, legible imaging acquisition. Secondary outcomes included the frequency and types of ECMO-associated ABI and a comparison of its detection by MR images compared to HCT images.

### Statistical Analysis

Descriptive statistical analysis was performed. Continuous variables were expressed as medians with interquartile range (IQR). Categorical variables were expressed as frequencies with percentages. Wilcoxon rank-sum and Pearson’s chi-squared tests were used to compare continuous and categorical variables, respectively. Demographic and clinical variables including ECMO variables in subjects with and without ABI were compared. P values < 0.05 were considered statistically significant. All analyses were carried out in STATA 17 (StataCorp, LLP, College Station, TX).

## Results

### Patient Characteristics

Based on the inclusion criteria, 53 patients were eligible for a scan and 3 patients could not be scanned due to facial/scalp edema and large head size that did not fit with the head coil of the ULF-pMRI scanner. Thus, ULF-pMRI was performed in 50 patients (median = 58 years; 52% males), 34 (68%) with VA-ECMO and 16 (32%) with VV-ECMO. Of 34 VA-ECMO patients, 11 (32% of 50) were centrally cannulated and 23 (68%) were peripherally cannulated. In 16 VV-ECMO patients, 9 (56%) had single lumen cannulation and 7 (44%) had double lumen cannulation. The studied patients had a median GCS of 6 and Sequential Organ Failure Assessment (SOFA) of 13 at the time of ULF-pMRI. Baseline and clinical characteristics, including ECMO variables, of the cohort are summarized in Table 1.

### Safety of ULF-pMRI

All patients were imaged successfully with ULF-pMRI, achieving the primary outcomes in 2 centers. AEs occurred in 3 (6%) patients (Table 2, **Supplemental Table 2**) with 2 minor AEs (4%) and 1 serious AE (2%). An ECMO suction event (minor AE, related) occurred in one patient (2%) due to insufficient support of the thoracolumbar spine leading to flexion at the groin. Repositioning the body, avoiding flexion of the spine between the bed and the scanning platform, resolved this. The ULF-pMRI scan was resumed without further issues. The second minor AE event was a transient decrease in ECMO flow from 6.0 to 0.9 L/min, accompanied by 4% decrease in MAP for several minutes. The patient recovered spontaneously. This AE was deemed unrelated to pMRI as the patient had experienced a similar event earlier in the day, well before the scan. One (2%) serious AE deemed related to the scan when, 6 minutes into the scan, the patient’s intra-aortic balloon pump (IABP) stopped functioning. The scan was paused. The IABP had been set to ECG trigger, and we suspected that the magnetic field generated by the pMRI system was affecting the patient’s ECG leads, causing significant artifacts. The IABP trigger was then switched to internal trigger at a set rate of 75 beats per minute, and the scan was resumed and completed successfully without any hemodynamic issues **(Supplemental Fig. 1)**.

### Validation of ULF-pMRI

All patients received ULF-pMRI exams at the patient’s bedside with T1-weighted, T2-weighted, FLAIR, and DWI with ADC map sequences. The average time from ECMO cannulation to pMRI was 3 days (Table 2). Overall, pMR images demonstrated good quality images to discern intracranial pathologies with good quality, assessed by the board-certified neuroradiologists. Eight patients (16%) had imaging artifacts (**Supplemental Table 3**). The artifacts were mostly regional and did not influence the ability to diagnose ABI, except for one case (2%) that had a significant artifact which impacted the accurate assessment of intracranial pathology. This artifact was noted only on DWI sequence at the skull base and the imaging resolution was suboptimal, with an uncertainty if the observed abnormality represented infarct vs. artifact since it encompassed different vascular territories (Fig. 2).

### Incidence and Type of ABI

Of 50, 22 (44%) had ABI during ECMO support in ULF-pMRI scans (Table 3, **Supplemental Table 4**). Ischemic stroke (n = 18, 36%) was the most common type of ABI, followed by ICH (n = 3, 6%) and HIBI (n = 2, 4%). A total of 36 patients (72%) had both pMRI and HCT exams during ECMO support, and 18 patients (36%) had both pMRI and HCT within 24 hours of each other. Of these 18 patients with both neuroimaging studies within 24 hours, 9 (50%) had both negative pMRI and HCT scans. ABI was observed in the remaining 9 patients (50%) with a total of 10 events (8 ischemic, 2 hemorrhagic events (ICH)). Of the 8 ischemic events, pMRI observed all 8, while HCT only observed 4 events. For ICH, pMRI observed only, while HCT observed both (2 events).

## Discussion

Our study demonstrated the safety and validity of an ULF-pMRI system in patients with ECMO support, which is an external validation of our first case series of human subjects.^10^ ULF-pMRI was first shown to be beneficial in neurocritical care ICU patients as an innovative neuroimaging system at the bedside with good sensitivity and specificity for diagnosing ABI^7^; ischemic stroke and ICH detection for pMRI were 100%^11^ and 80%^12^ in non-ECMO patients. We previously demonstrated the safety and compatibility of ULF-pMRI in ECMO without deleterious magnetic force (no displacement of the cannula) or heating of the ECMO circuit or its components in a phantom model and 3 human subjects.^9^ Now based on the results of our multicenter prospective cohort study, we externally validated the safety of ULF-pMRI in 50 patients on ECMO support. This data is important not only for ECMO patients but also for those with other cardiac devices in whom ≥ 1.5T MRI is contraindicated where ULF-pMRI has the potential to change our neurological diagnostic approach. As timely and accurate ABI diagnosis is crucial in improving the overall ECMO outcomes,^13^ ULF-pMRI can eliminate the need to transport unstable ECMO patients, which has the potential to universally reshape clinical care and outcomes and ECMO clinical trials with the promise of accurately diagnosing ABI promptly.

### Adverse Events

We reported 2 minor AEs (ECMO suction event and a transient decrease in ECMO flow) in our prior publication in 3 patients,^10^ which was likely related to the initial learning curve in scanning ECMO patients with pMRI. After optimizing our workflow and the logistics of performing pMRI in ECMO patients (i.e., optimal patient positioning: positioning the patient’s head and body as flat as possible, preventing flexion of the spine between the bed and the scanning platform^10^), we only had 1 AE in 47 patients between 2 centers. One (2%) serious AE (related) occurred when the IABP stopped functioning as a result of being set to ECG trigger with the magnetic field causing significant ECG artifacts on telemetry. Although IABP trigger was switched to an internal set rate (internal-trigger) in our case, in patients who are pulsatile, pressure-trigger would be preferred as an internal set rate could inflate the balloon during systole and adversely affect left ventricular unloading.

### Incidence and Type of ABI

**Figure 1:**
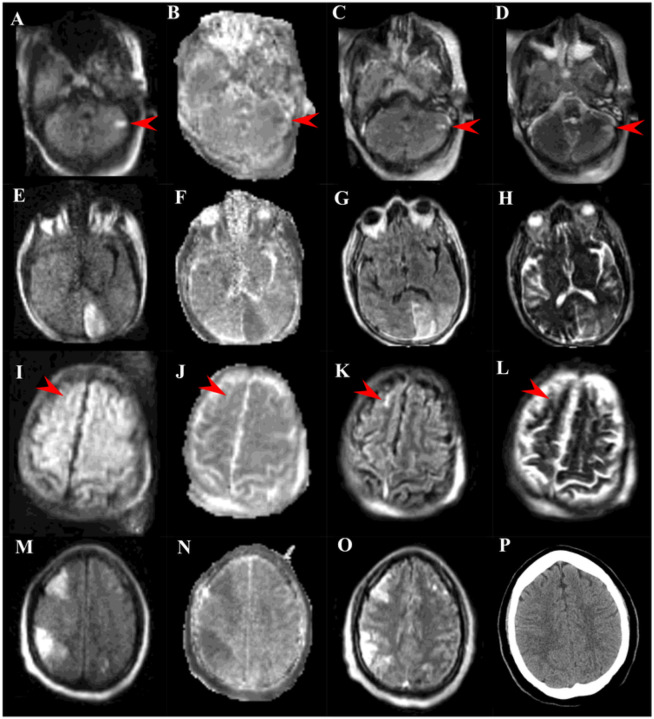
Ultra-low-field portable MRI (ULF-pMRI) images and head CT image in patients with extracorporeal membrane oxygenation (ECMO) support. Images **A**, **E**, **I**, **M** are diffusion-weighted imaging (DWI) MRI, images **B**, **F**, **J**, **N** are apparent diffusion coefficient MRI, images **C**, **G**, **K**, **O** are fluid-attenuated inversion recovery (FLAIR) MRI, images **D**, **H**, **L** are T2-weighted MRI, and image **P** is a CT scan. Patient 1 (Images **A–D**) demonstrated small ischemic stroke in the left cerebellum denoted by red arrows. Patient 2 (**E–H**) demonstrated moderate ischemic stroke in the left occipital lobe. Patient 3 (**I–L**) had an intracranial hemorrhage in the right superior frontal sulcus (red arrow). Patient 4 (**M-O**) demonstrated multifocal infarcts in the bilateral superior frontal and parietal lobes on MRI images and had a normal CT scan. Entire series of MRI sequences and images for these patients are available as the link to Digital Imaging and Communications in Medicine (DICOM) in the Hyperfine, Inc. Imaging Viewer.

**Figure 2:**
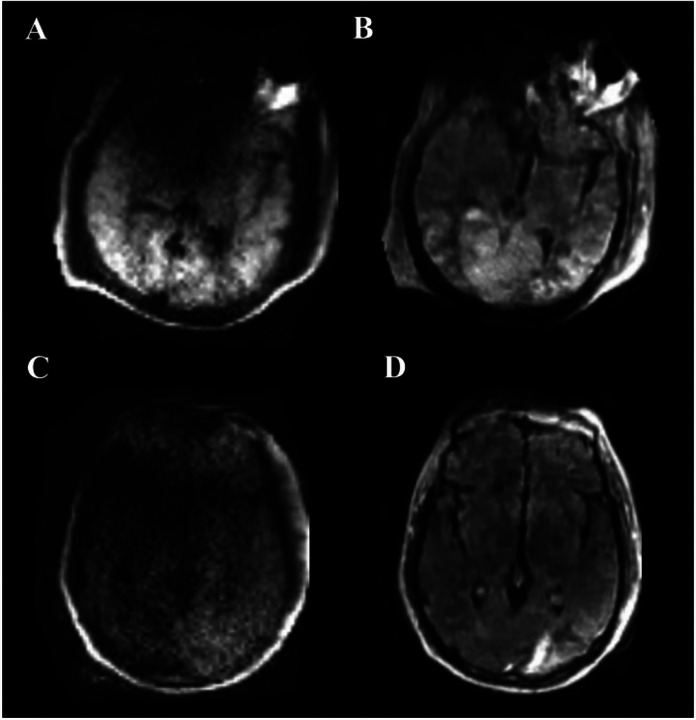
ULF-pMRI images showing artifacts. Overall, DWI MRI (Images **A** and **C**) depict artifacts with greater clarity, while FLAIR MRI (**B** and**D**) shows fewer artifacts, relatively. Patient 1 (**A-B**) had an ischemic stroke in the left temporal and both occipital lobes, and Patient 2 (**C-D**) in the left occipital lobe. Patient 1 has a regional minor artifact anteriorly, obscuring anterior inferior brain, but it does not impact the diagnosis. Patient 2 has a major artifact in the area of the right cerebral hemisphere in general, leading to uncertainty about whether it represents infarct or artifact.

We previously demonstrated that the application of the standardized noninvasive neuromonitoring protocol including HCT led to an expected increase in early ABI detection with 33% ABI frequency.^5,13^ Therefore, our study confirmed our hypothesis that ULF-pMRI detects ABI (44%) better than the conventional approach utilizing HCT. Notably, ULF-pMRI was able to detect even small- to moderate-sized ischemic infarcts, which otherwise would have not been possible to detect in HCT (Fig. 1). Also, it’s important to note that ULF-pMRI likely is not able to detect tiny “punctate” infarcts that often are visualized in conventional MRI. When comparing ULF-pMRI to HCT in 18 patients who had both, ULF-pMR outperformed HCT for the detection of ABI, especially ischemic injury, and may underperform for hemorrhagic injury (Table 4, **Supplemental Fig. 2**). The suboptimal sensitivity to ICH, although a small sample size, may improve with modification of the imaging acquisition technology.

Of note, the standardized neuromonitoring protocol was associated with a significant improvement in favorable neurological outcomes (modified Rankin Scale ≤ 3) in ECMO survivors compared to the era before the standardized neuromonitoring protocol (33% vs. 14%).^5,13^ This highlights the importance of adding ULF-pMRI to the standardized neuromonitoring approach, as early accurate ABI detection and early interventions are critical for mitigating ABI (i.e., anticoagulation management) and worsening neurological outcomes.

### Potential Benefits in ECMO Clinical Trials

**Figure 3:**
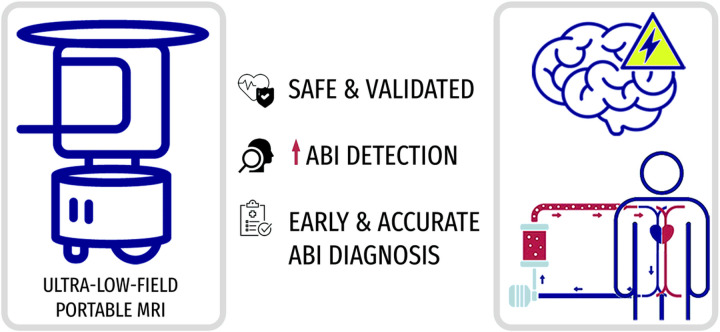
ULF-pMRI allows early and accurate diagnosis of acute brain injury (ABI) that can lead to migitation strategy for extracorporeal membrane oxygenation (ECMO) associated ABI.

Recent randomized controlled trials (RCTs) on extracorporeal cardiopulmonary resuscitation (ECPR) patients (ARREST,^13^ PRAGUE,^14,15^ and INCEPTION^16^) and cardiogenic shock patients (ECLS-SHOCK^17^; albeit 77% of included patients had pre-ECMO cardiac arrest) trials demonstrated that ABI is the leading cause of death and ECMO discontinuation in VA-ECMO patients, which most likely contributed to the neutral trial results in survival outcomes. The unfavorable neurological prognosis (43%) was the leading cause of ECMO discontinuation in the INCEPTION trial,^16^ and 25% of deaths were due to brain death in the PRAGUE trial in the ECPR group.^14,15^ Although ABI is a major factor, if not the leading cause, for poor survival outcomes in the ECMO RCTs, there is significant heterogeneity and a lack of standardization in ABI definitions and post-ECMO neurological approach and neurocritical care. Our study suggests that ULF-pMRI, as a standard neuroimaging diagnostic tool, can be used in future clinical trials (standardization, Fig. 3).

### Limitations

Our study has several limitations. First, the timing of ULF-pMRI and HCT were not mandated in the study. Although we provide information on the comparison between two neuroimaging modalities, only 36% (n = 18) had both imaging studies within 24 hours. Furthermore, ongoing injuries can occur within several hours between the scans, which cannot be captured in our study. However, we took a pragmatic approach as moving “unstable” ECMO patients for another neuroimaging scan always carries a risk during transport. Second, we were not able to provide information on “symptomatic” vs. “asymptomatic” brain lesions as most of these patients are heavily sedated early in the ECMO course and, thus, it is difficult to draw a meaningful conclusion whether all observed ABIs were clinically significant. However, it is also important to highlight the fact that any “silent” ABI is highly likely associated with long-term outcomes such as cognitive function.^18^ Because neurological exams are often not feasible or sensitive in the presence of critical illness and sedative use in ECMO, the results of this study provide an insight as to how to improve future ECMO clinical trial design. Lastly, other cardiac devices such as percutaneous ventricular assist devices or left ventricular assist devices are often placed along with ECMO and these patients were excluded from this trial as the safety was not proven. Pre-clinical and clinical safety were demonstrated in IABP and pMRI.^19^

## Conclusions

ULF-pMRI is shown to be safe in ECMO patients across different ECMO cannulation strategies. The incidence of ABI was high with 44% in ULF-pMRI, with ischemic stroke being the most common type. Our study, by introducing and testing an alternative form of neuroimaging, will significantly lower this barrier to diagnosing and treating ECMO-associated ABI. This innovative approach can inform the design of subsequent clinical trials and validate strategies to mitigate ECMO-associated ABI.

## Figures and Tables

**Table 1:** Baseline and clinical characteristics of ECMO patients.

	Total (n = 50)	Patients without ABI (n = 28)	Patients with ABI (n = 22)	P value
Demographics				
Age, years	58 (41.3–69)	59 (40.2–69)	56.5 (42.5–69.2)	0.89
Male	26 (52%)	18 (64.3%)	8 (36.4%)	0.09
Body Mass Index, kg/m^2^	31.2 ±7.0	30.0 ±5.7	32.8 ±8.3	0.17
Race				
White	22 (44%)	12 (42.9%)	10 (45.5%)	1.00
Black	17 (34%)	11 (39.3%)	6 (27.3%)	0.56
Hispanic	5 (10%)	2 (7.1%)	3 (13.6%)	0.64
Asian	3 (6%)	2 (7.1%)	1 (4.5%)	1.00
Others	3 (6%)	1 (3.6%)	2 (9.1%)	0.58
**Past medical history**				
Ischemic stroke	6 (12%)	2 (7.1%)	4 (18.2%)	0.38
Intracranial hemorrhage	2 (4%)	1 (3.6%)	1 (4.5%)	1.00
Hypertension	27 (54%)	15 (53.6%)	12 (54.5%)	1.00
Hyperlipidemia	25 (50%)	13 (46.4%)	12 (54.5%)	0.78
Diabetes mellitus	20 (40%)	11 (39.3%)	9 (40.9%)	1.00
Heart failure	13 (26%)	8 (28.6%)	5 (22.7%)	0.89
Chronic kidney disease	18 (36%)	11 (39.3%)	7 (31.8%)	0.80
Atrial fibrillation	11 (22%)	8 (28.6%)	3 (13.6%)	0.31
**PreECMO variables**				
Inotropic or vasoactive support	40 (80%)	22 (78.6%)	18 (81.8%)	1.00
Cardiac arrest	11 (22%)	5 (17.9%)	6 (27.2%)	0.50
Glasgow Coma Scale	6 (3–13)	6 (3–13)	6 (3–12)	0.75
SOFA score (on MRI day)	13 (11–15)	13 (9.8–14)	13 (11–16)	0.52
**ECMO Cannulation**				
VA-ECMO				
Central cannulation	11 (22%)	7 (25%)	4 (18.2%)	0.73
Peripheral cannulation	23 (46%)	13 (46.4%)	10 (45.5%)	1.00
VV-ECMO				
Single lumen cannulation	9 (18%)	4 (14.3%)	5 (22.7%)	0.48
Double lumen cannulation	7 (14%)	4 (14.3%)	3 (13.6%)	1.00
**Mortality**	31 (62%)	18 (64.3%)	13 (59%)	0.93
**ECMO duration (days)**	9 (4.3–21.5)	10 (5.8–17.8)	8 (4–25)	0.38
VA-ECMO duration (days)	8.5 (4–12.5)	9 (3.8–11.5)	8 (4.8–19.2)	0.75
VV-ECMO duration (days)	24 (7.8–38.8)	33.5 (17.8–52.5)	7.5 (3.8–28.5)	0.03

ECMO: extracorporeal membrane oxygenation; VA: venoarterial; VV: venovenous; ABI: acute brain injury; SOFA: Sequential Organ Failure Assessment.

**Table 2:** Time from cannulation to ULF-pMRI and the frequency of adverse events during the scan.

Time from ECMO to Imaging	Total (n = 50)	No ABI (n = 28)	ABI (n = 22)	P-value
Average time from ECMO cannulation to pMRI (days)	3.0 (1.0–5.0)	3.0 (1–5.3)	3.5 (1.3–4.8)	0.77
Average time from ECMO cannulation to HCT (days)	2.0 (0–6.3)	2.0 (0–7.0)	2.0 (1.0–4.0)	0.69
Adverse events during pMRI	Total (n = 50)	No ABI (n = 28)	ABI (n = 22)	P-value
All adverse events	3 (6%)	0	3 (13.6%)	NA
Minor adverse events	2 (4%)	0	2 (4%)	NA
Serious adverse events	1 (2%)	0	1 (2%)	NA

ULF-pMRI: ultra-low-field portable MRI; HCT: head CT; NA: not applicable.

**Table 3:** Incidence and type of acut brain injury (ABI) in ULF-pMRI.

Type of ABI	ABI Frequency (n, %)
Ischemic stroke	18 (36%)
Hypoxic ischemic brain injury	2 (4%)
Intracranial hemorrhage	3 (6%)
Intraparenchymal hemorrhage	1 (2%)
Subarachnoid hemorrhage	1 (2%)
Subdural hemorrhage	1 (2%)
Brain death	2 (4%)
**Patients with ABI**	**22 (44%)**

ULF-pMRI: ultra-low-field (0.064T) portable MRI; ABI: acute brain injury.

**Table 4:** In 18 patients in whom both a HCT and ULF-pMRI were obtained, the breakdown of MRI and HCT diagnosis in the 10 patients with ABI.

ABI			
Ischemic (8 events)		Hemorrhagic (2 Events)	
MRI (+)	CT (+)	MRI (+)	CT (+)
8 (100%)	4 (50%)	1 (50%)	2 (100%)

ULF-pMRI: ultra-low-field (0.064T) portable MRI; HCT: head CT; ABI: acute brain injury.

## Take-home message

- Ultra-low-field (0.064T) portable MRI (ULF-pMRI) is safe in patients on extracorporeal membrane oxygenation (ECMO) support across different ECMO cannulation strategies.
- The incidence of ABI was high, seen in 44% of ULF-pMRI, studies, with ischemic stroke comprising the vast majority. ULF-pMRI imaging appears to be more sensitive to ABI, particularly ischemic stroke, when compared the CT.
